# Supplementary material for: Classification of RNA backbone conformations into rotamers using 13C′ chemical shifts: exploring how far we can go
Source: PeerJ. 2019 Oct 21;7:e7904. doi: 10.7717/peerj.7904 (PMC6812668; doi:10.7717/peerj.7904)
Supplement: Supplemental Information 1 [file peerj-07-7904-s001.pdf]

Supplementary Data

Classification of RNA backbone conformations into rotamers using $^{13}C'$ chemical shifts: How far we can go?

1 Experimental database

PDB ID	BMRB ID
1AJU	15869
1HWQ	5007
1KKA	5256
1NC0	5655
1Q75	5932
1R7W	6076
1YSV	6485
1ZC5	6633
2JPP	15257
2KOC	5705
2KZL	17316
2LU0	18503
2M22	18892
2M4W	19024
2MEQ	18975
2MFC	19544
2MFF	19547
2MFG	19548
2MHI	19634
2N7X	25826
2N82	25831
2O32	15080
2QH2	7403
2QH3	7404
2QH4	7405
5KQE	30132

Table S1: BMRB and PDB IDs for the 26 RNA structures of the experimental dataset.

2 Distribution of rotamers in the experimental database

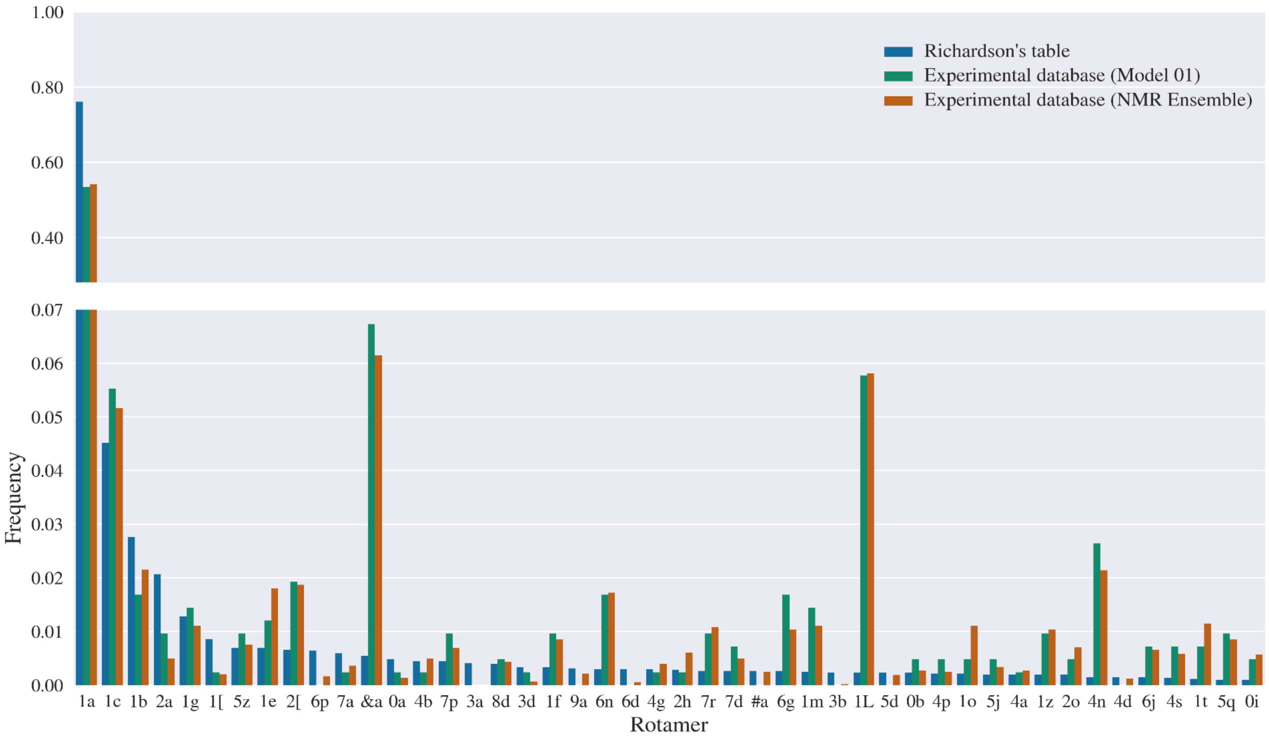

Supplementary Figure S1: Observed frequency of rotamers as reported by Richardson [1] (blue bars), as calculated from the first model in the experimental NMR ensembles (green bars) and from the experimental NMR ensembles as a whole (orange bars). A broken y-axis is used in order to show two different frequency range scales.

3 Construction of the ROSUM matrices

The jk-element of the ROSUM matrix (denoted as a_{jk}) is defined by Equation 1, where P_{jk} is the probability of substitution of rotamer j (i.e. true rotamer) by rotamer k (i.e. predicted rotamer), and it is defined by Equation 2. The probability of substituion P_{jk} is obtained from the normalized distance D_{norm} (Equations 3 and 4), where distance D_{jk} is the sum of the intra–rotameric distances d_{jj} and d_{kk}, and the inter–rotameric distance d_{jk}. An intra–rotameric distance is the average of the torsion angle standard deviations of a given rotamer (Equations 5 and 6). The inter–rotameric distance is the euclidean distance between the torsion angles mean values of rotamer j and rotamer k (Equation 7). Both the torsion angles mean values and the standard deviations were extracted from the Richardson's rotamer table [1].

$$a_{jk} = log(\frac{P_{jk}}{q_j * q_k}) \tag{1}$$

$$P_{jk} = \frac{1}{D_{norm}} \tag{2}$$

$$D_{norm,s} = \frac{D_{jk}}{\sum D_{jk}} \tag{3}$$

$$D_{jk} = d_{jj} + d_{kk} + d_{jk} \tag{4}$$

$$d_{rr} = \frac{\sqrt{\sum (\sigma_{x_r})^2}}{7} \tag{5}$$

where:

$rr = jj$ for true rotamer intra–rotameric distance or $rr = kk$ for predicted rotamer intra–rotameric distance; x are the torsionals (δ_{i-1}, ε_{i-1}, ζ_{i-1}, α_i, β_i, γ_i, δ_i) in true rotamer j or predcited rotamer k, respectively; and the constant in the denominator is the number of torsionals in a rotamer suite, seven.

$$d_{jk} = \sqrt{\sum (x_j - x_k)^2} \tag{6}$$

where:

x are the torsionals (δ_{i-1}, ε_{i-1}, ζ_{i-1}, α_i, β_i, γ_i, δ_i) in rotamer j and rotamer k

When j and k are rotamer families, the frequencies q_j and q_k are the sum over the observed frequencies of the rotamer members of families j and k, respectively, and D_{norm} is the average distance between the rotamer members of family j and family k.
Below, we show a representation of the ROSUM matrices. The figure sizes are conserved to illustrate how rotamers are being grouped into families containing an increasing number of rotamers.

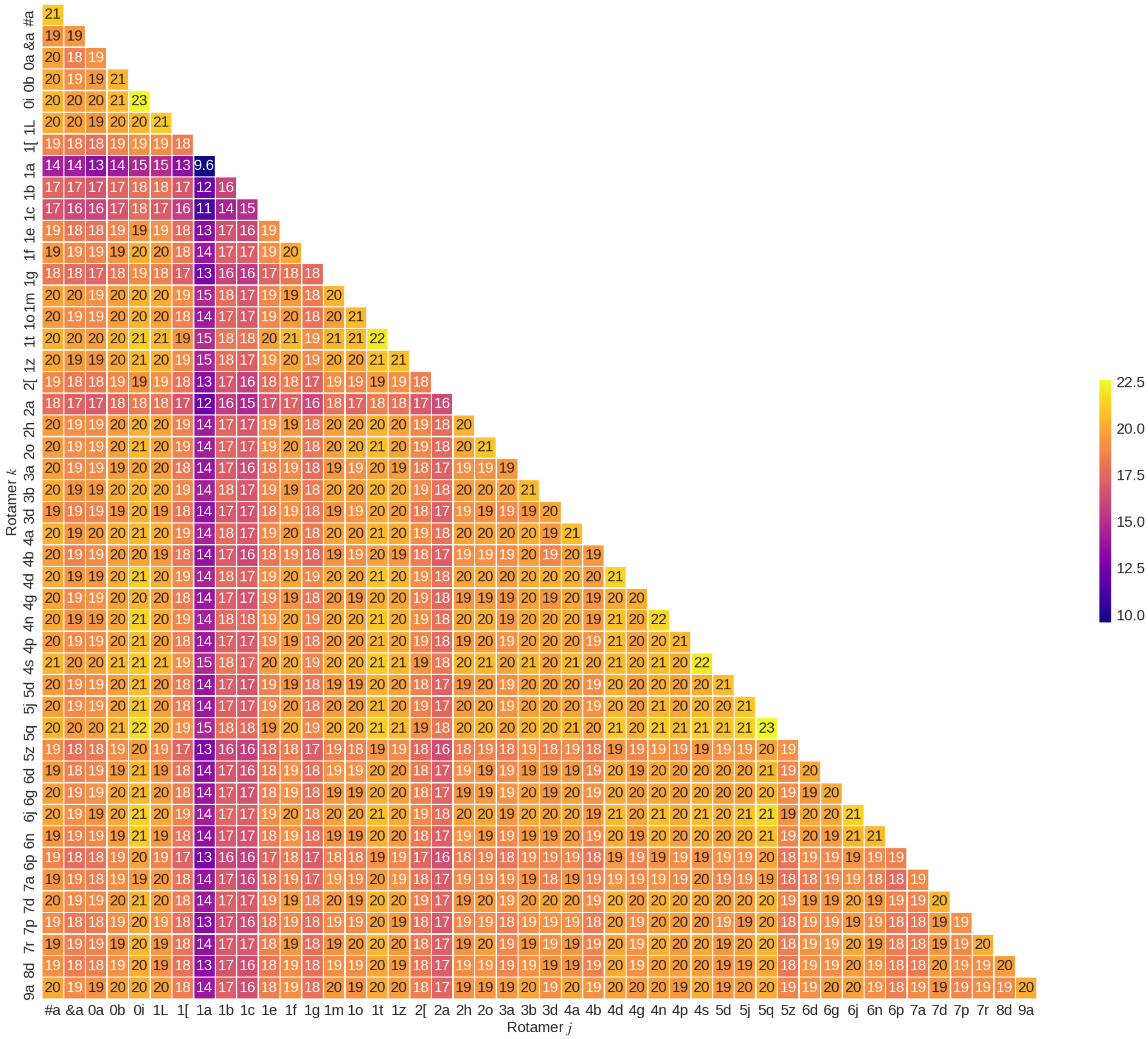

Figure S2: Representation of the ROSUM matrix for the original 46 rotamers.

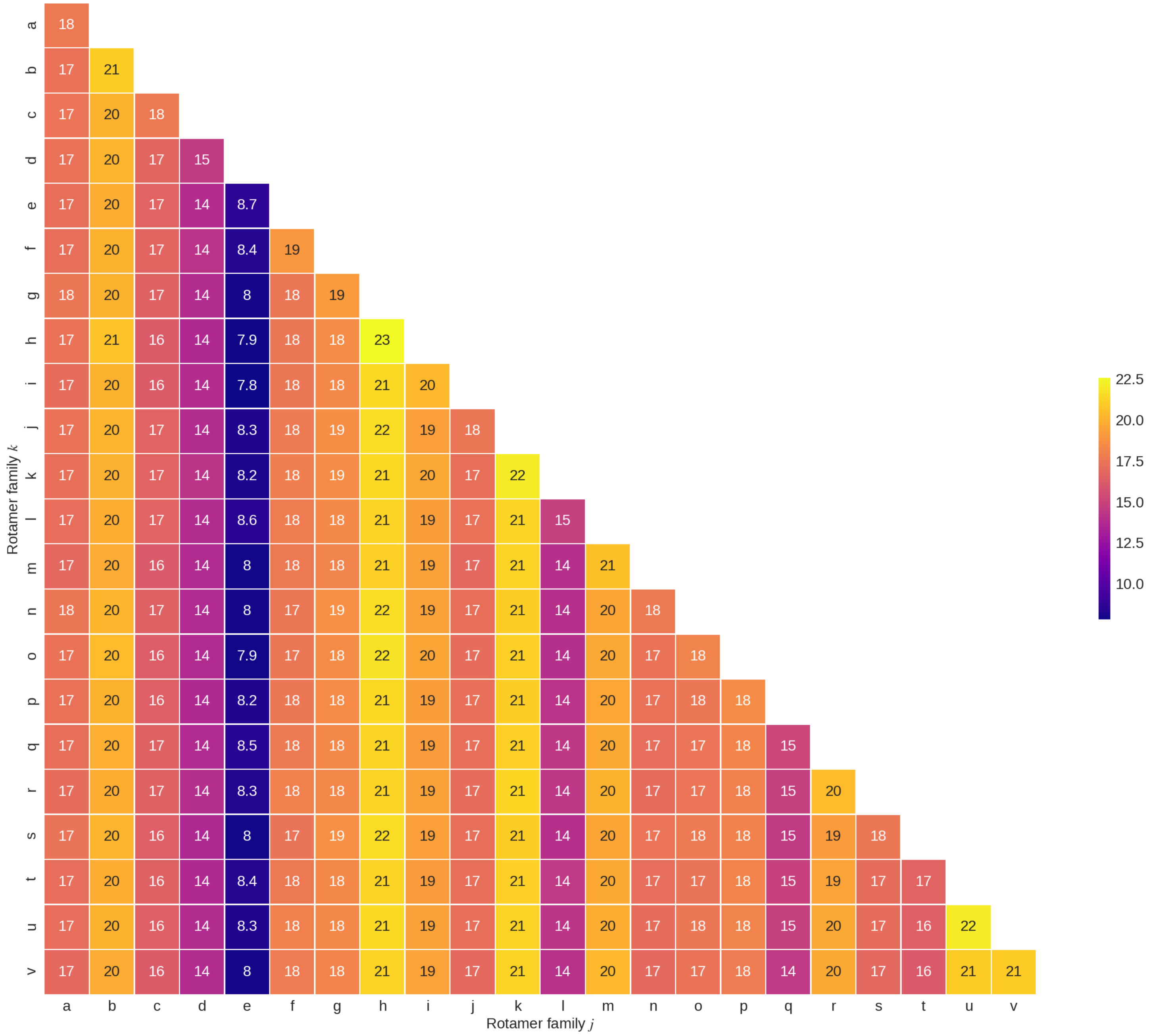

Figure S3: Representation of the ROSUM matrix for $\delta_{i-1}\delta_i\alpha\gamma$ families.

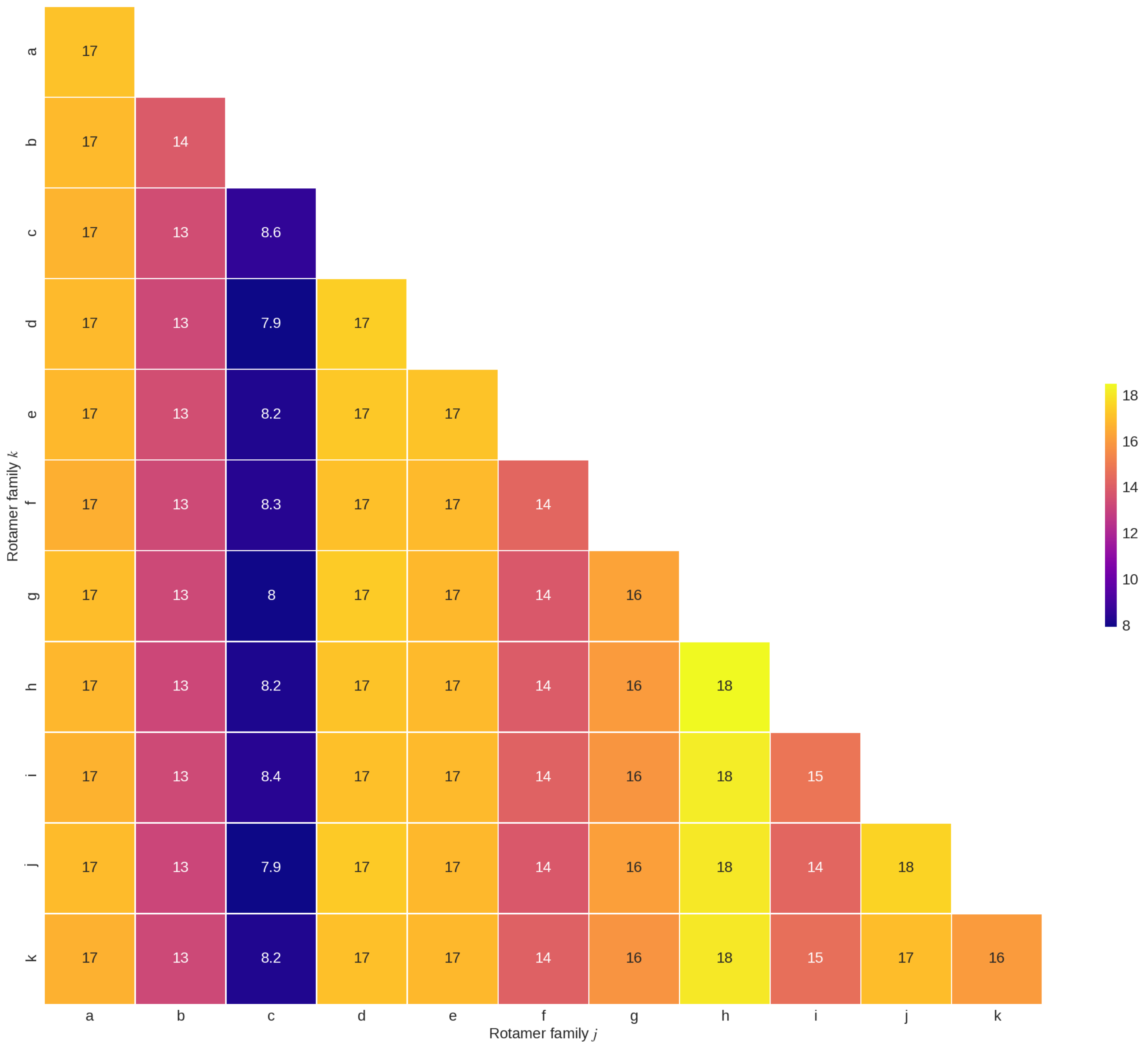

Figure S4: Representation of the ROSUM matrix for $\delta_{i-1}\delta_i\alpha$ families.

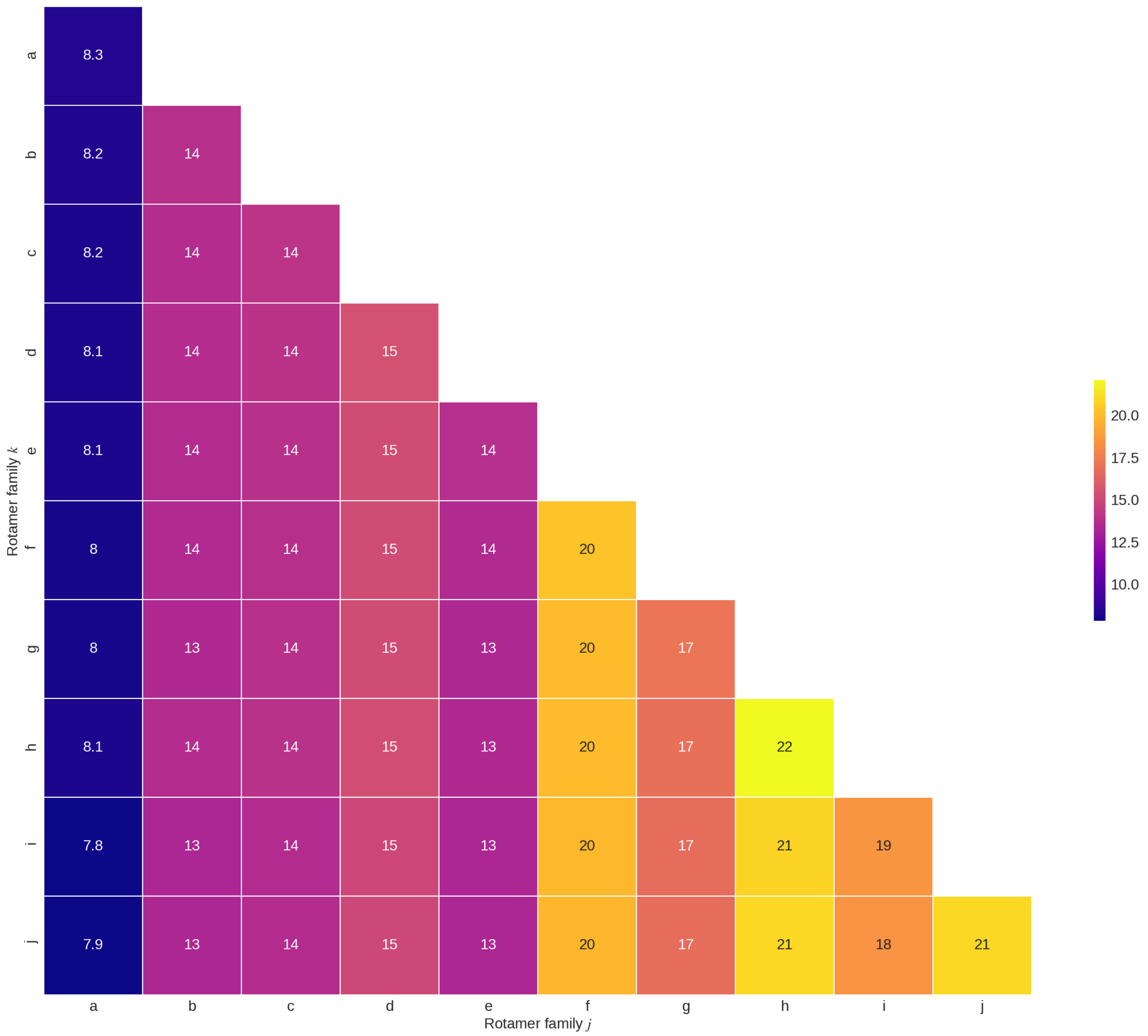

Figure S5: Representation of the ROSUM matrix for $\delta_{i-1}\delta_i\gamma$ families.

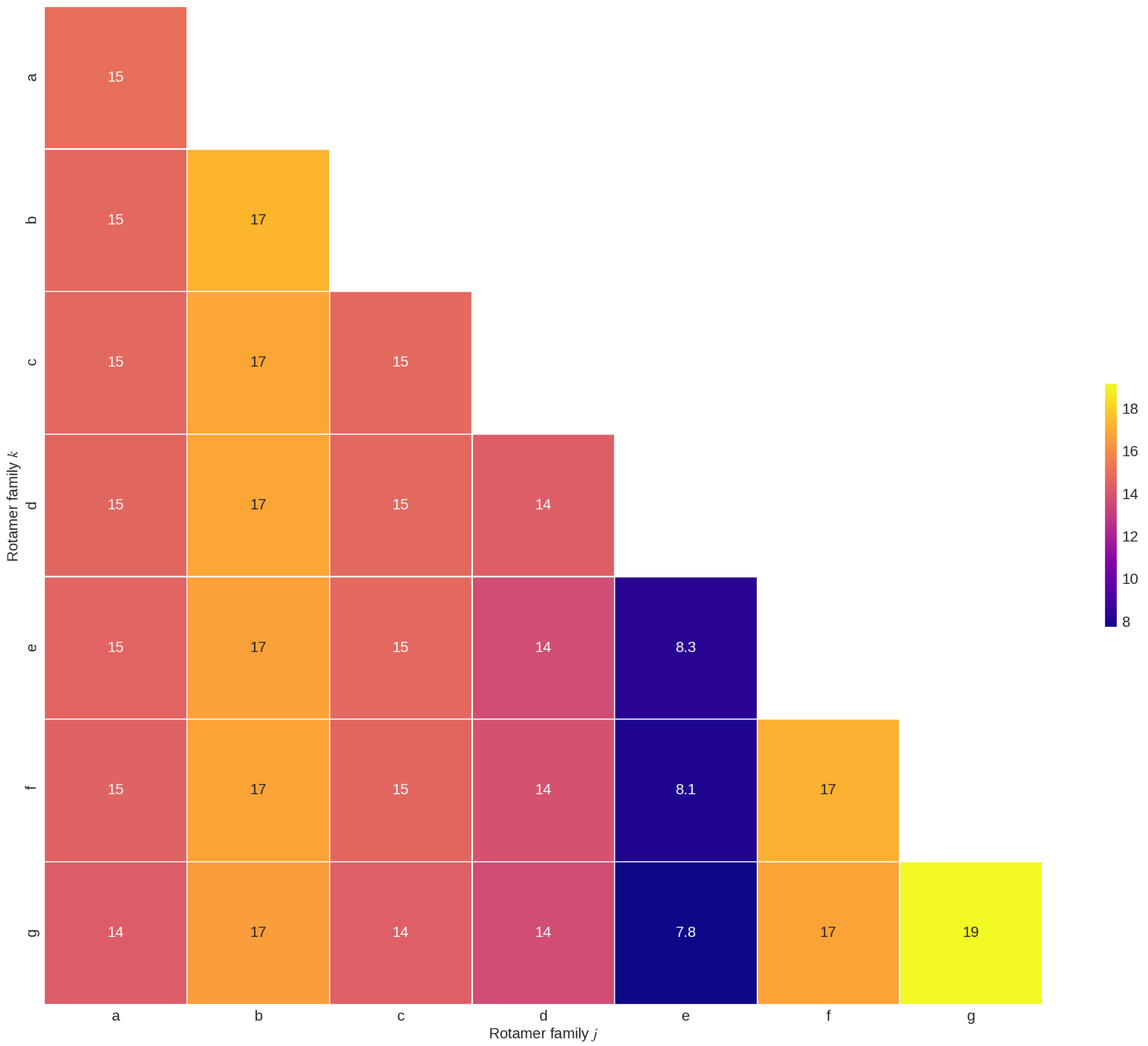

Figure S6: Representation of the ROSUM matrix for $\alpha\gamma$ families.

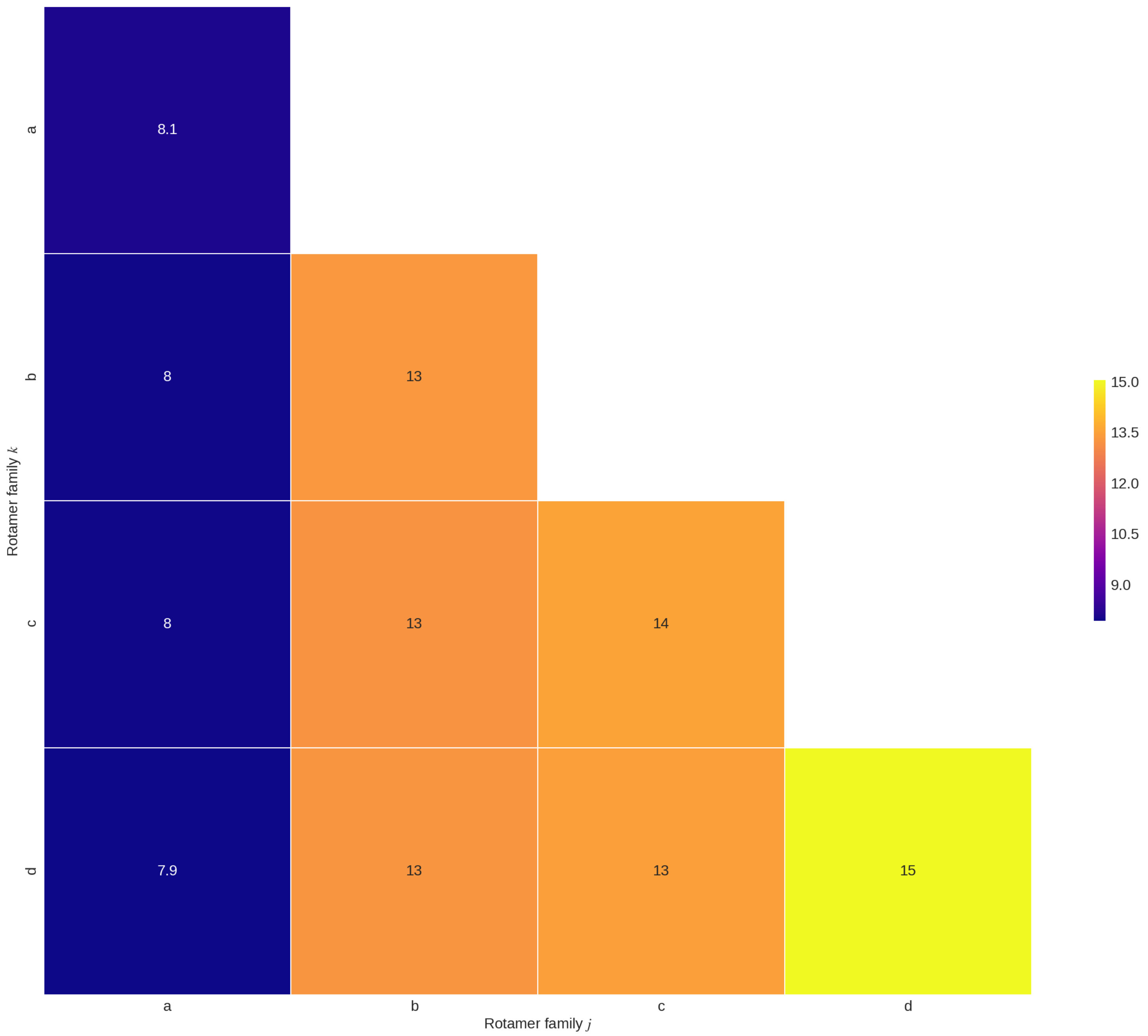

Figure S7: Representation of the ROSUM matrix for $\delta_{i-1}\delta_i$ families.

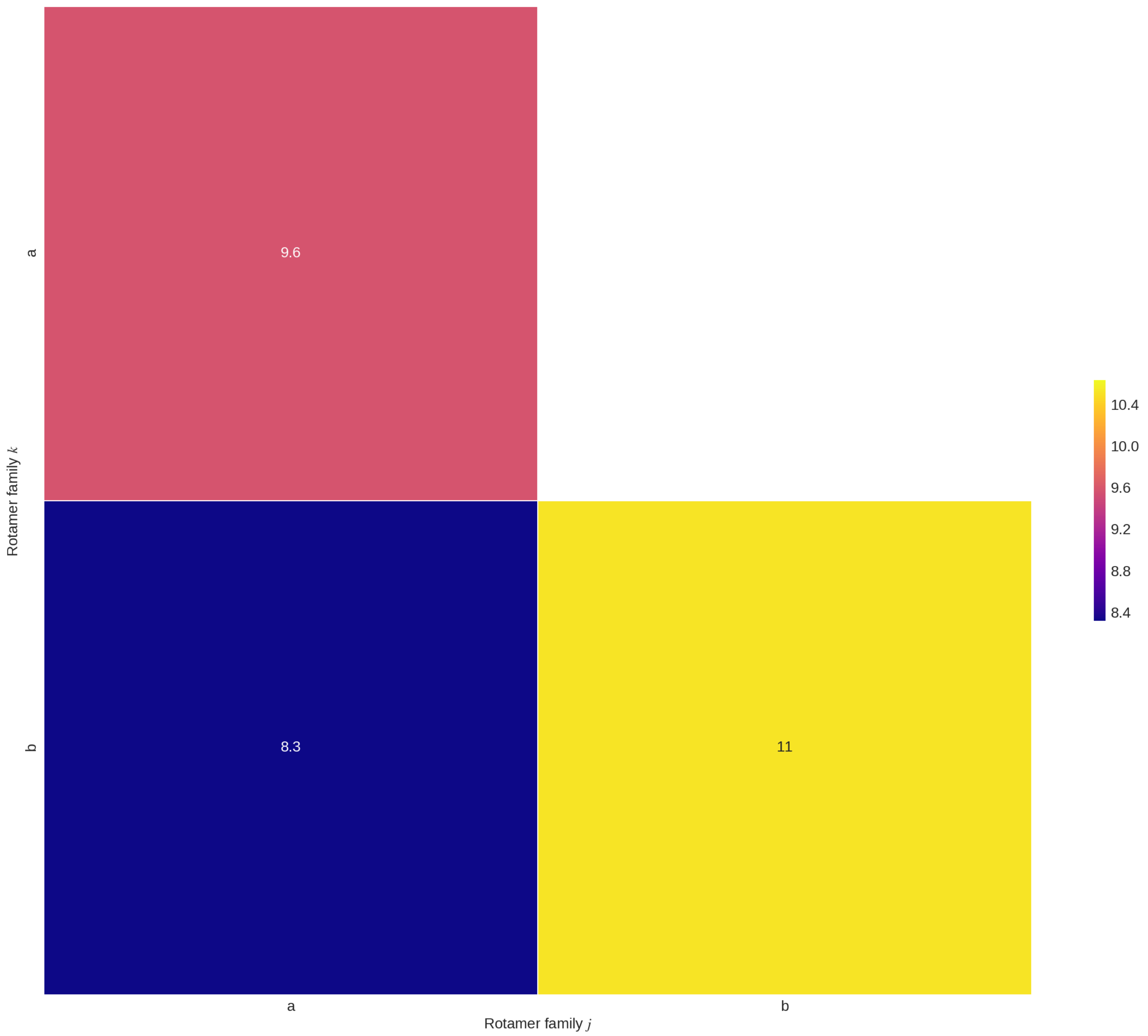

Figure S8: Representation of the ROSUM matrix for A_noA families.

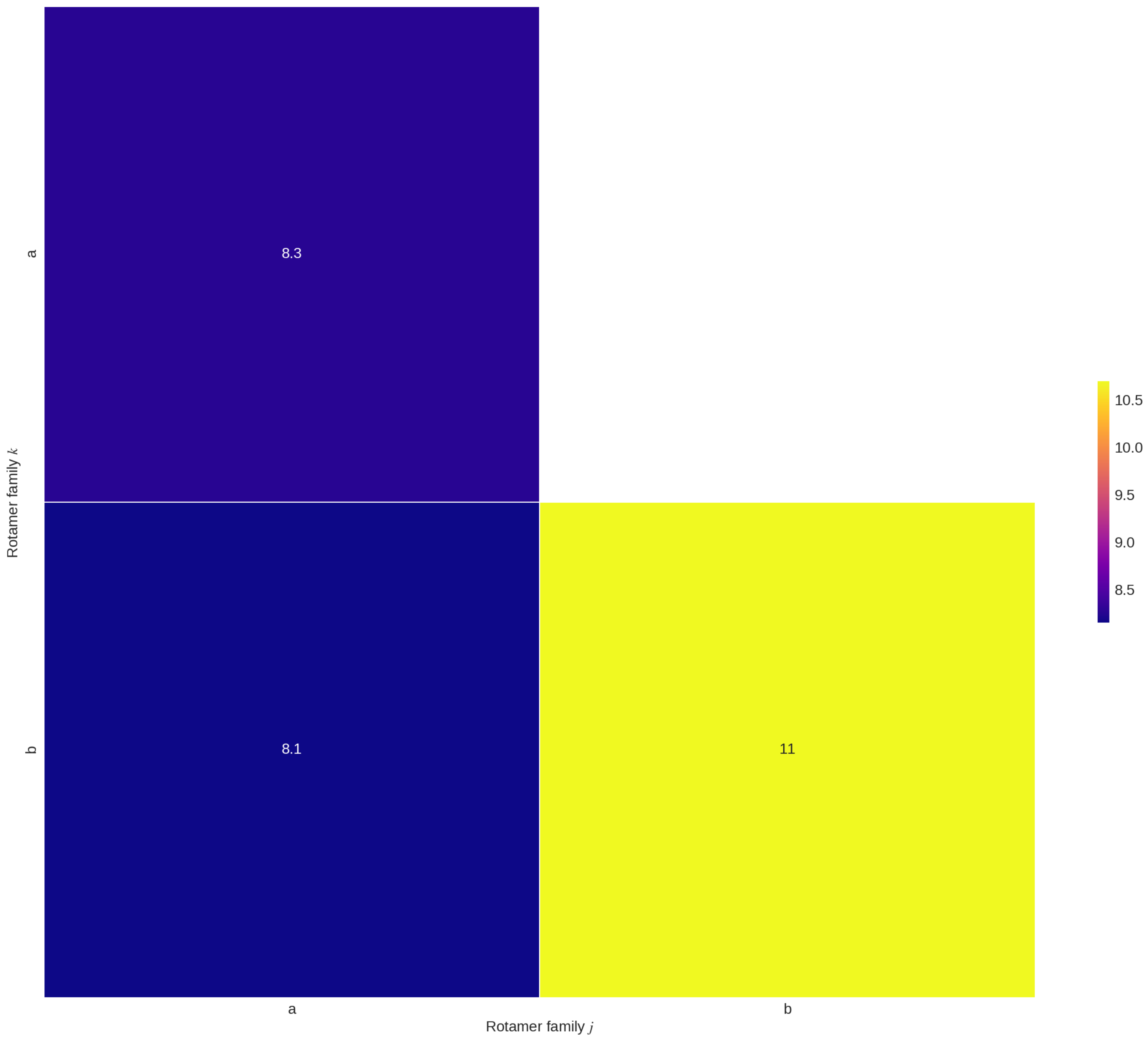

Figure S9: Representation of the ROSUM matrix for A*_noA* families.

4 Effective references

Reference type	Value
**Simple**	185.0
**Sequence**	
AA	181.5
AC	183.5
AG	185.0
AU	185.0
CA	185.0
CC	180.0
CG	187.9
CU	180.0
GA	185.0
GC	185.8
GG	185.2
GU	184.2
UA	180.0
UC	185.0
UG	189.7
UU	185.0
**Puckering**	
C3'endo-C3'endo	183.9
C3'endo-C2'endo	181.2
C2'endo-C3'endo	182.9
C2'endo-C2'endo	181.6
**C_mean**	
C1'i-1	183.04
C2'i-1	179.30
C3'i-1	179.91
C4'i-1	181.76
C5'i-1	183.06
C1'i	182.67
C2'i	179.23
C3'i	180.30
C4'i	182.06
C5'i	183.36
**C_opt**	
C1'	a= 74.75, b= 0.19
C2'	a= 70.58, b= 0.06
C3'	a= 56.64, b= 0.22
C4'	a= 63.79, b= 0.23
C5'	a= 53.75, b= 0.18

Table S2: Effective references used to transform the theoretical $^{13}C'$ shieldings into theoretical $^{13}C'$ chemical shifts. The **Sequence** and **Puckering** labels correspond to the reference values that gave the highest performance in experimental vs theoretical classification using the 1-NN model, for the 16 dinucleotide sequences and the 4 combinations of ribose puckering, respectively. The **C_mean** values were obtained by comparing the mean value of the distribution of experimental $^{13}C'$ chemical shifts and the mean value of the distribution of theoretical $^{13}C'$ shieldings, for the ten different nuclei in a dinucleotide (or suite). For **C_opt**, the values of a and b in the equation $\delta_{comp} = a + b{*}\sigma_{comp}$ for the five different carbon nuclei in a nucleotide are given. Here, δ_{comp} and σ_{comp} refer to theoretical chemical shifts and theoretical shieldings, respectively. The values of a and b were obtained trough a hierarchical model of a linear regression using PyMC3 [2] for a set of dinucleotides with theoretical and experimental $^{13}C'$ chemical shifts.

5 Classification models

5.1 Scikit-learn parameters

Model	Parameters
RandomForestClassifier	(criterion='gini', max_depth=20, n_estimators=10, max_features=1)
RandomForestClassifier	(criterion='gini', max_depth=20, n_estimators=10, max_features='auto')
RandomForestClassifier	(criterion='entropy', max_depth=20, n_estimators=10, max_features=1)
RandomForestClassifier	(criterion='entropy', max_depth=20, n_estimators=10, max_features='auto')
DecisionTreeClassifier	(criterion='gini', max_depth=20)
DecisionTreeClassifier	(criterion='gini', max_depth=None)
DecisionTreeClassifier	(criterion='entropy', max_depth=20)
DecisionTreeClassifier	(criterion='entropy', max_depth=None)
SVC	(kernel='linear', C=0.025)
SVC	(kernel='linear', C=0.1)
SVC	(kernel='linear', C=0.5)
SVC	(kernel='linear', C=1.0)
SVC	(kernel='linear', C=2.0)
SVC	(kernel='rbf', C=0.025)
SVC	(kernel='rbf', C=0.1)
SVC	(kernel='rbf', C=0.5)
SVC	(kernel='rbf', C=1.0)
SVC	(kernel='rbf', C=2.0)
KNeighborsClassifier	(k=1)
KNeighborsClassifier	(k=2)
KNeighborsClassifier	(k=3)
KNeighborsClassifier	(k=4)
KNeighborsClassifier	(k=5)
MLPClassifier	(alpha=0.0001, max_iter=500, solver='lbfgs')
MLPClassifier	(alpha=0.0001, max_iter=500, solver='sgd')
MLPClassifier	(alpha=0.0001, max_iter=500, solver='adam')
MLPClassifier	(alpha=0.0001, max_iter=500, solver='sgd')
MLPClassifier	(alpha=0.0001, max_iter=750, solver='sgd')
MLPClassifier	(alpha=0.0001, max_iter=1000, solver='sgd')

Table S3: Sci-kit learn models and parameters used for classification.

6 Use of nucleotide sequence for classification

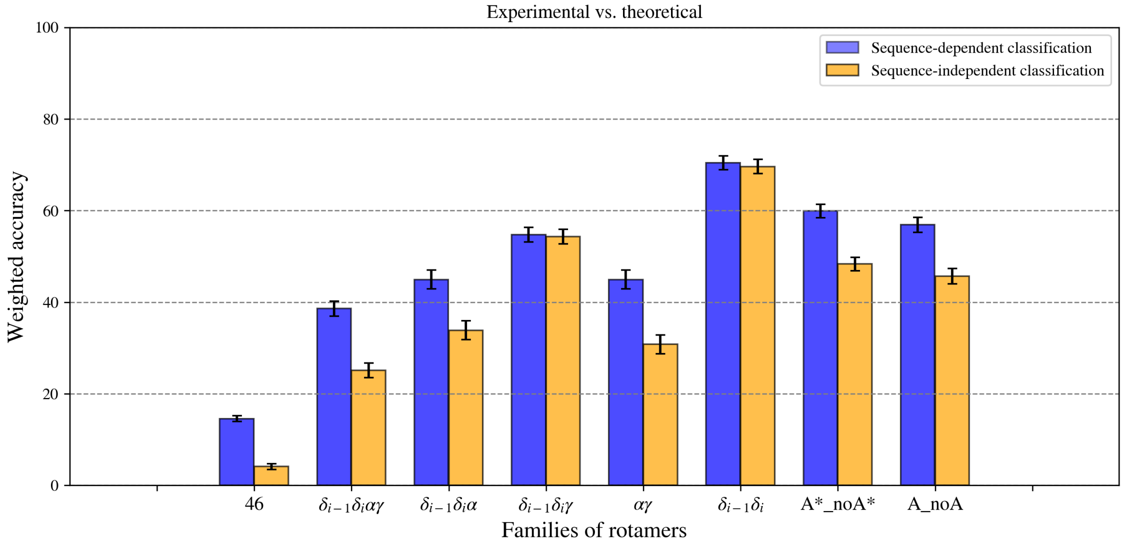

Supplementary Figure S10: Weighted accuracies for sequence-dependent and sequence-independent classifications, for the experimental dataset against the theoretical dataset, using a 1-NN model. Groups labels A_noA, A*_noA* and 46 indicate A–form helix rotamer (1a) vs no A–form helix rotamers, A–form helix related rotamers (1a, 3d, 3b, 5d, 0a, 6b and 4b) vs no A–form helix related rotamers, and the 46 original rotamers. While families labels $\delta_{i-1}\delta_i$, $\alpha\gamma$, $\delta_{i-1}\delta_i\alpha$, $\delta_{i-1}\delta_i\gamma$, $\delta_{i-1}\delta_i\alpha\gamma$ indicate the combination of torsional angles used for grouping. Blue and orange bars indicate sequence–dependent and sequence–independent classifications, respectively. Error bars indicate the standard deviation in the weighted accuracies over the conformations of the NMR assemblies.

7 Classification using all the models in the experimental NMR ensemble: experimental vs. theoretical classification

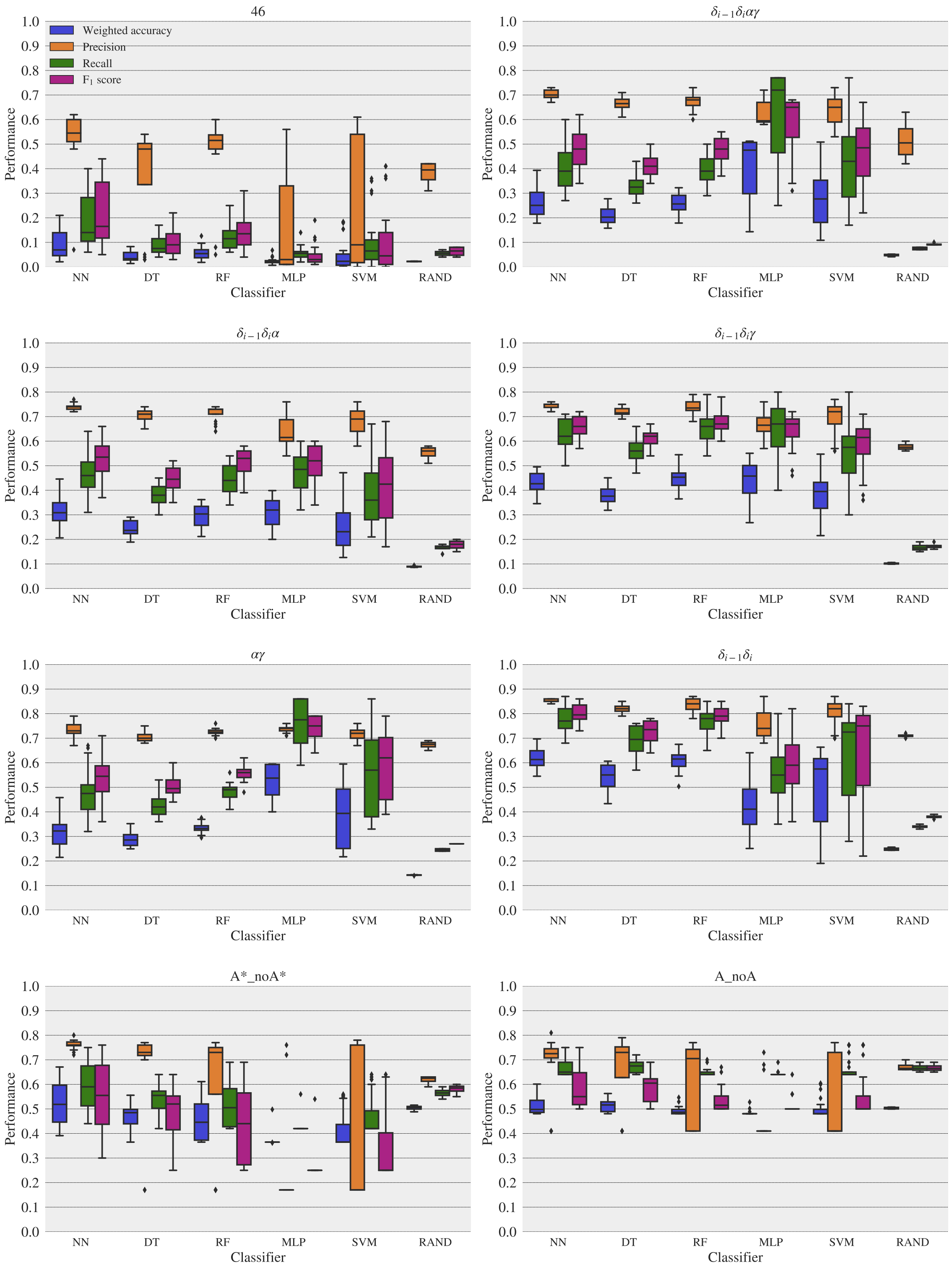

Figure S11: Box-plots with the four measures of performance (weighted accuracy, precision, recall and F_1 score) for the experimental vs. theoretical classification of rotamers and families of rotamers, using Nearest Neighbour (NN), Decision Tree (DT), Random Forest (RF), Multi-Layer Perceptron (MLP) and Support Vector Machine (SVM) classifiers. A random-choice (RAND) algorithm was used as a baseline reference. In this case, the predicted rotamer was compared against a list of rotamers that describe the suites in all the models from the experimental NMR ensemble.

8 Classification results displaying the four measures of performance: weighted accuracy, precision, recall and F_1 score

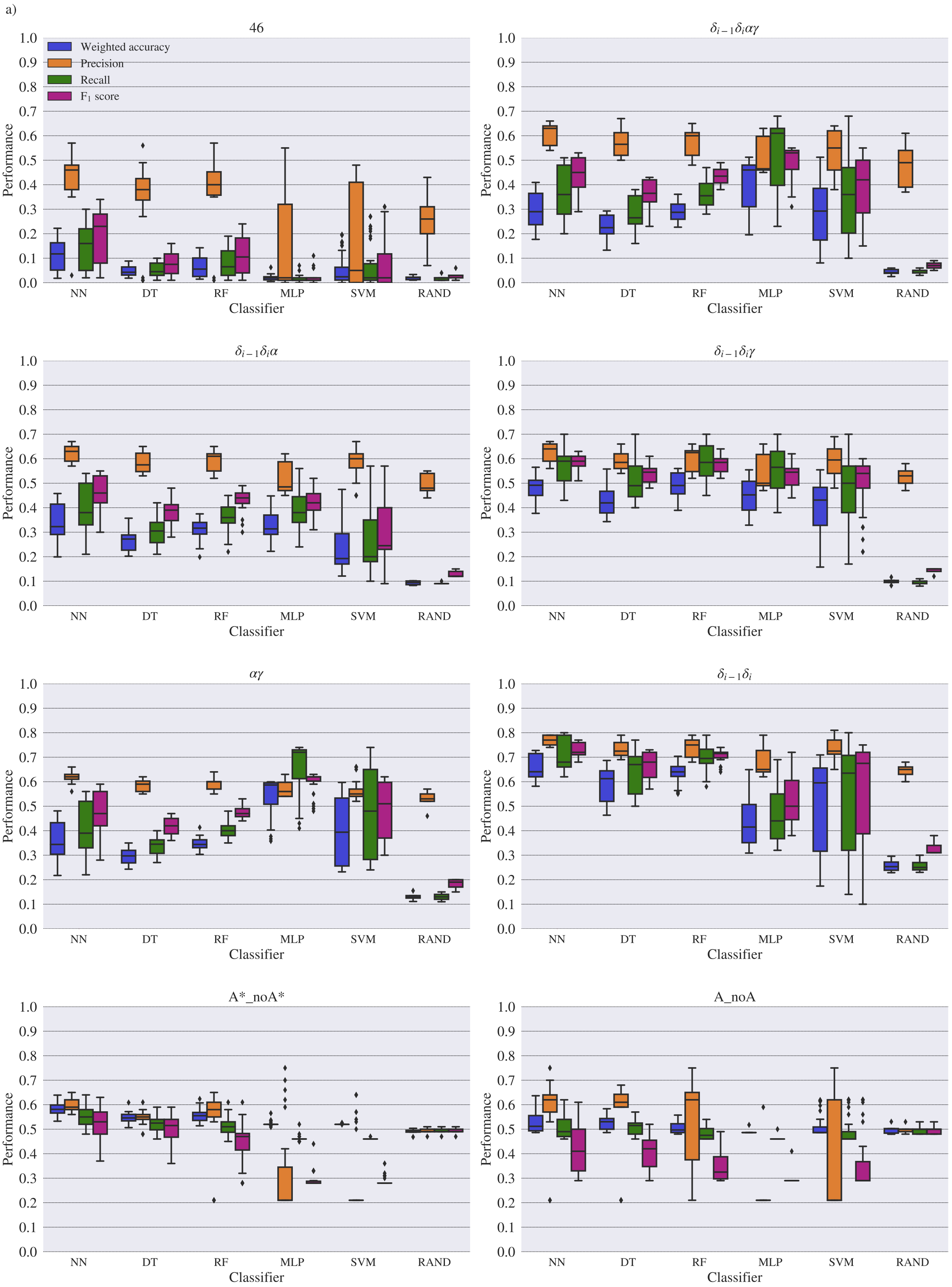

b)

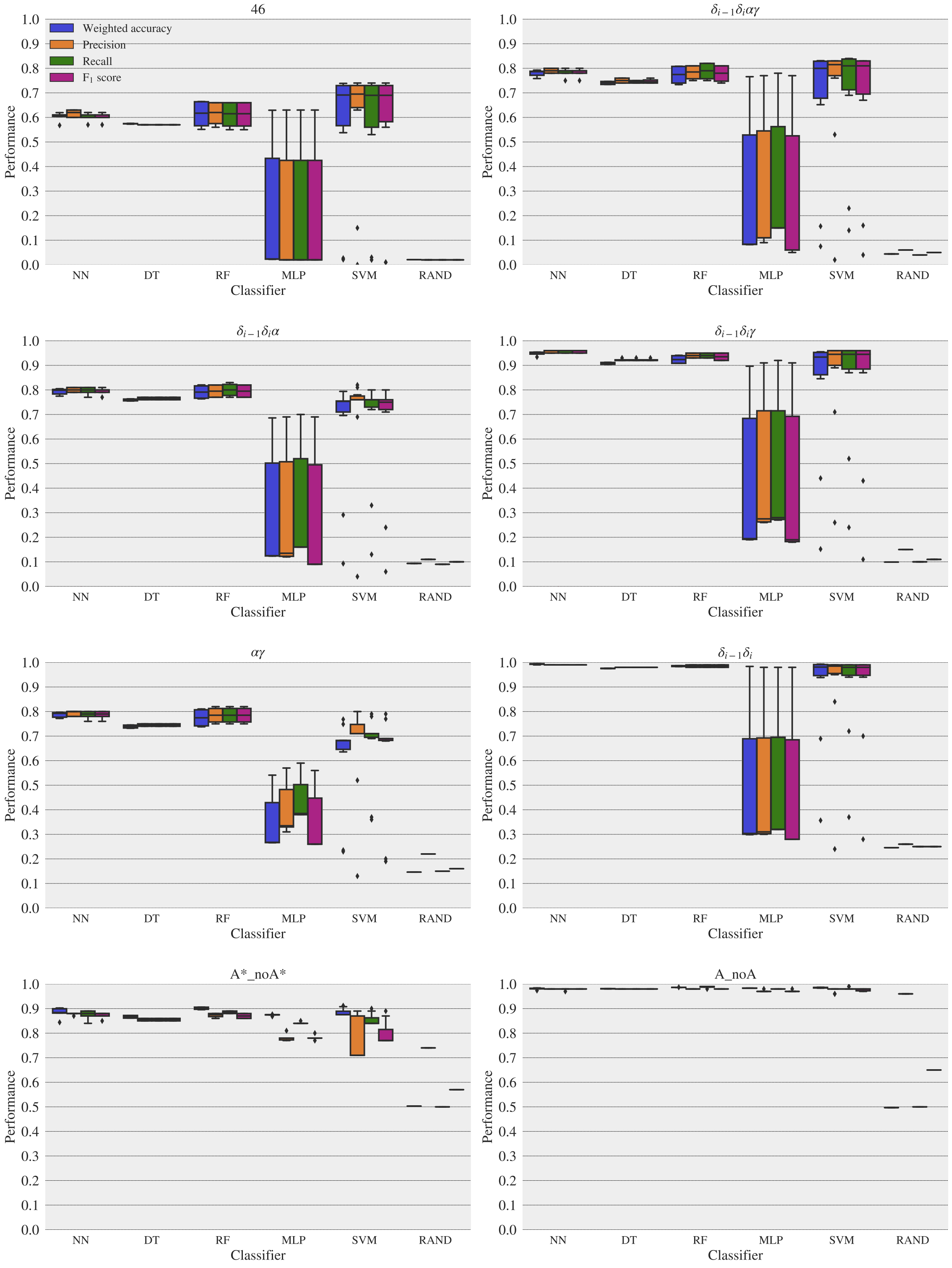
46
$\delta_{i-1}\delta_i\alpha\gamma$
$\delta_{i-1}\delta_i\alpha$
$\delta_{i-1}\delta_i\gamma$
$\alpha\gamma$
$\delta_{i-1}\delta_i$
A*_noA*
A_noA
Weighted accuracy
Precision
Recall
F_1 score
Performance
Classifier
NN
DT
RF
MLP
SVM
RAND
0.0
0.1
0.2
0.3
0.4
0.5
0.6
0.7
0.8
0.9
1.0

c)

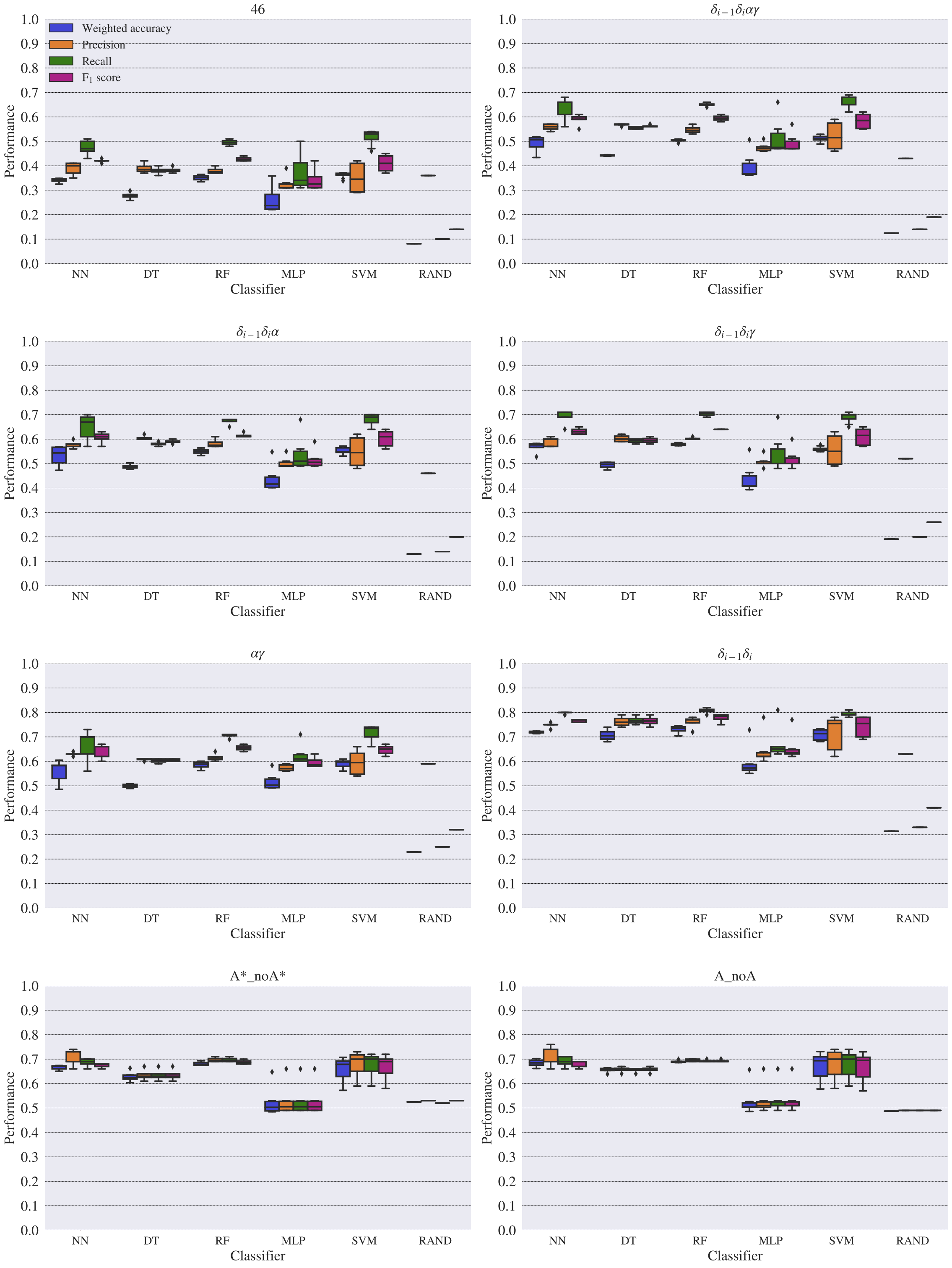

Figure S12: Box-plots with the four measures of performance (weighted accuracy, precision, recall and F_1 score) for the classification of rotamers and families of rotamers, using Nearest Neighbour (NN), Decision Tree (DT), Random Forest (RF), Multi-Layer Perceptron (MLP) and Support Vector Machine (SVM) classifiers. A random-choice (RAND) algorithm was used as a baseline reference. In this case, for experimental data, only the first model from the NMR ensemble was used. In a), the classification models were generated from theoretical data and were used to classify the experimental data. The results from theoretical vs theoretical classification and experimental vs experimental classification are shown in b) and c), respectively.

9 Theoretical vs. theoretical classification using a theoretical subset mimicking the sparsity of the experimental dataset

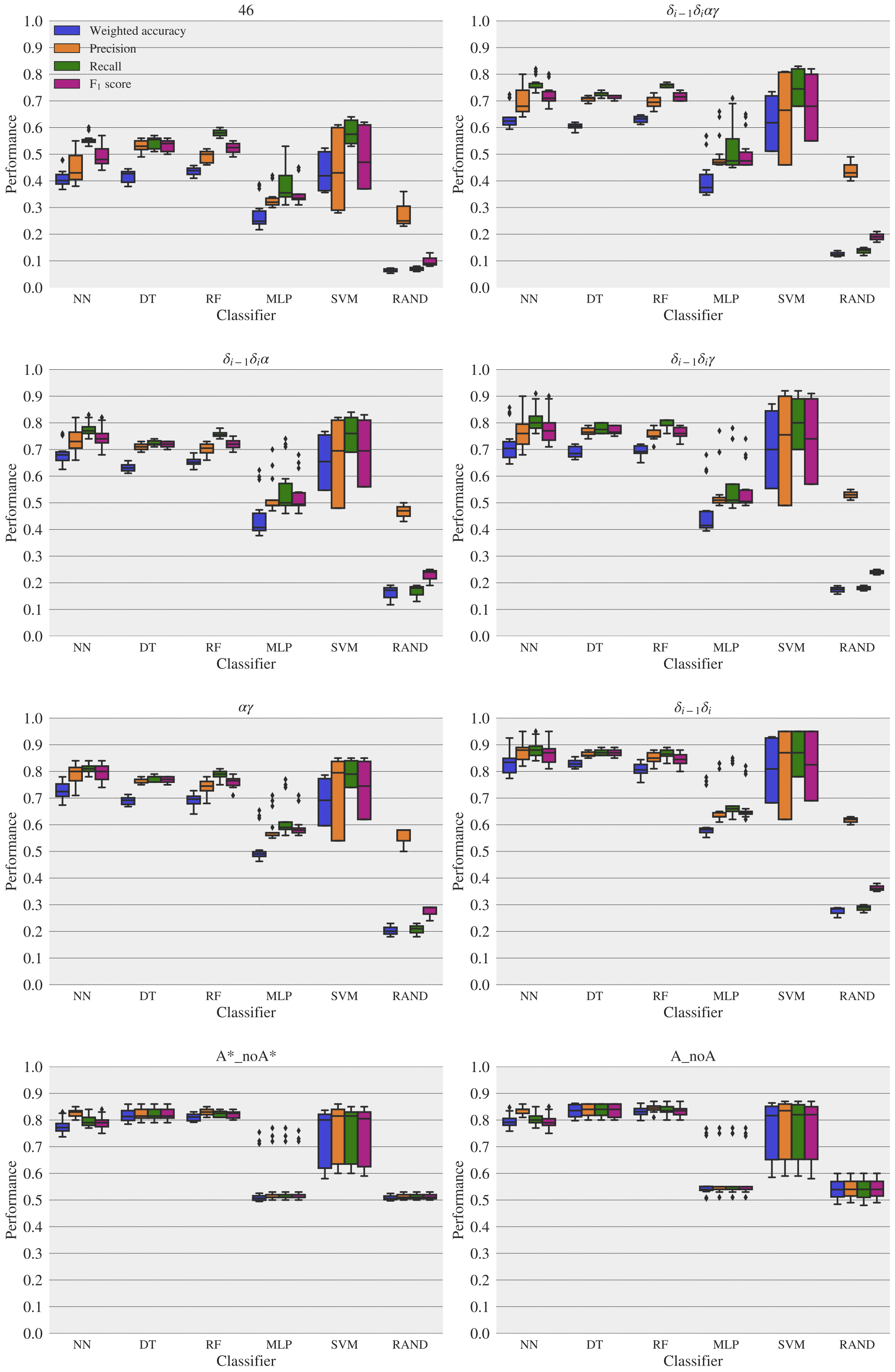

Figure S13: Box-plots with the four measures of performance (weighted accuracy, precision, recall and F_1 score) for the theoretical vs. theoretical classification of rotamers and families of rotamers, using Nearest Neighbour (NN), Decision Tree (DT), Random Forest (RF), Multi-Layer Perceptron (MLP) and Support Vector Machine (SVM) classifiers. A random-choice (RAND) algorithm was used as a baseline reference. In this case, elements were removed from the theoretical dataset in order to mimic the sparsity of the experimental dataset.

10 Theoretical vs. theoretical + noise classification

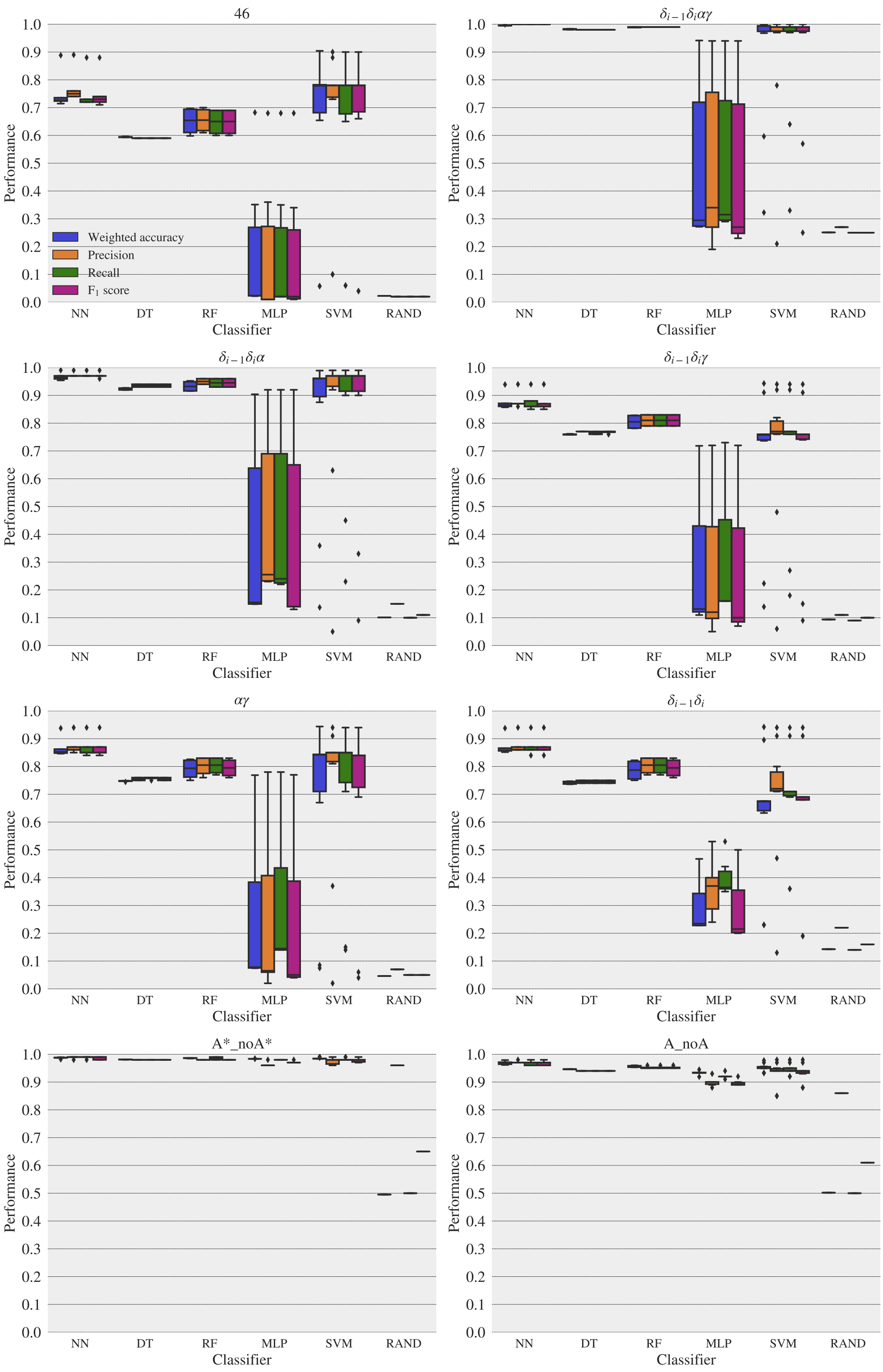

Figure S14: Box-plots with the four measures of performance (weighted accuracy, precision, recall and F_1 score) for the theoretical vs. theoretical + noise classification of rotamers and families of rotamers, using Nearest Neighbour (NN), Decision Tree (DT), Random Forest (RF), Multi-Layer Perceptron (MLP) and Support Vector Machine (SVM) classifiers. A random-choice (RAND) algorithm was used as a baseline reference. Noise, on the order of the expected error between experimental and theoretical $^{13}C'$ CS for those rotamers correctly classified, was added to the theoretical $^{13}C'$ CS and then a theoretical vs. theoretical + noise classification was performed.

10.1 Scikit-learn parameters for the highest scores of experimental vs theoretical classification

Table S4: Scikit–learn classifiers parameters for the highest values of weighted accuracy.

Rotamer families	Reference[a]	Classifier[a]	Scikit-learn parameters	Weighted accuracy
46	Sequence	NN	KNeighborsClassifier(2)	0.22
$\delta_{i-1}\delta_i\alpha\gamma$	Sequence	NN	KNeighborsClassifier(4)	0.41
$\delta_{i-1}\delta_i\alpha$	Simple	NN	KNeighborsClassifier(4)	0.46
$\delta_{i-1}\delta_i\gamma$	Simple	NN	KNeighborsClassifier(2)	0.57
$\alpha\gamma$	Sequence	NN	KNeighborsClassifier(4)	0.48
$\delta_{i-1}\delta_i$	Sequence	NN	KNeighborsClassifier(2)	0.73
A*_noA*	Sequence	NN	KNeighborsClassifier(1)	0.64
A _noA	Puckering	NN	KNeighborsClassifier(2)	0.64
46	Sequence	DT	DecisionTreeClassifier(criterion='entropy', max_depth=20)	0.09
$\delta_{i-1}\delta_i\alpha\gamma$	Sequence	DT	DecisionTreeClassifier(criterion='entropy', max_depth=20)	0.29
$\delta_{i-1}\delta_i\alpha$	Simple	DT	DecisionTreeClassifier(criterion='gini', max_depth=None)	0.36
$\delta_{i-1}\delta_i\gamma$	Simple	DT	DecisionTreeClassifier(criterion='entropy', max_depth=None)	0.56
$\alpha\gamma$	Sequence	DT	DecisionTreeClassifier(criterion='entropy', max_depth=None)	0.35
$\delta_{i-1}\delta_i$	Simple	DT	DecisionTreeClassifier(criterion='entropy', max_depth=None)	0.69
A*_noA*	Sequence	DT	DecisionTreeClassifier(criterion='entropy', max_depth=20)	0.61
A _noA	Sequence	DT	DecisionTreeClassifier(criterion='entropy', max_depth=20)	0.58
46	Simple	RF	RandomForestClassifier(criterion='gini', max_depth=20, n_estimators=10, max_features=1)	0.14
$\delta_{i-1}\delta_i\alpha\gamma$	Simple	RF	RandomForestClassifier(criterion='entropy', max_depth=20, n_estimators=10, max_features=1)	0.36
$\delta_{i-1}\delta_i\alpha$	Simple	RF	RandomForestClassifier(criterion='entropy', max_depth=20, n_estimators=10, max_features=1)	0.37
$\delta_{i-1}\delta_i\gamma$	Simple	RF	RandomForestClassifier(criterion='entropy', max_depth=20, n_estimators=10, max_features=1)	0.56
$\alpha\gamma$	Sequence	RF	RandomForestClassifier(criterion='entropy', max_depth=20, n_estimators=10, max_features=1)	0.41
$\delta_{i-1}\delta_i$	Puckering	RF	RandomForestClassifier(criterion='gini', max_depth=20, n_estimators=10, max_features=1)	0.70
A*_noA*	Sequence	RF	RandomForestClassifier(criterion='entropy', max_depth=20, n_estimators=10, max_features='auto')	0.62
A _noA	Sequence	RF	RandomForestClassifier(criterion='entropy', max_depth=20, n_estimators=10, max_features='auto')	0.56
46	Puckering	MLP	MLPClassifier(alpha=0.0001, max_iter=500, solver='lbfgs')	0.06
$\delta_{i-1}\delta_i\alpha\gamma$	C_opt	MLP	MLPClassifier(alpha=0.0001, max_iter=750, solver='sgd')	0.51
$\delta_{i-1}\delta_i\alpha$	C_opt	MLP	MLPClassifier(alpha=0.0001, max_iter=1000, solver='sgd')	0.45
$\delta_{i-1}\delta_i\gamma$	Simple	MLP	MLPClassifier(alpha=0.0001, max_iter=1000, solver='sgd')	0.55
$\alpha\gamma$	Puckering	MLP	MLPClassifier(alpha=0.0001, max_iter=1000, solver='sgd')	0.60
$\delta_{i-1}\delta_i$	Simple	MLP	MLPClassifier(alpha=0.0001, max_iter=500, solver='adam')	0.65
A*_noA*	C_opt	MLP	MLPClassifier(alpha=0.0001, max_iter=500, solver='lbfgs')	0.57
A _noA	C_opt	MLP	MLPClassifier(alpha=0.0001, max_iter=500, solver='lbfgs')	0.52
46	Sequence	SVM	SVC(kernel='linear', C=0.025)	0.20
$\delta_{i-1}\delta_i\alpha\gamma$	Simple	SVM	SVC(kernel='rbf', C=0.025)	0.51
$\delta_{i-1}\delta_i\alpha$	Simple	SVM	SVC(kernel='rbf', C=0.025)	0.47
$\delta_{i-1}\delta_i\gamma$	Simple	SVM	SVC(kernel='rbf', C=0.025)	0.55
$\alpha\gamma$	Simple	SVM	SVC(kernel='rbf', C=0.025)	0.60
$\delta_{i-1}\delta_i$	Simple	SVM	SVC(kernel='linear', C=0.025)	0.71
A*_noA*	Simple	SVM	SVC(kernel='rbf', C=1.0)	0.53
A _noA	Simple	SVM	SVC(kernel='linear', C=0.5)	0.62

***a* Reference** refers to the effective references used to obtain the theoretical $^{13}C'$ chemical shifts (see Section 4).

***b* Classifier**: Nearest Neighbor (NN), Decision Tree (DT), Random Forest (RF), Multi-Layer Perceptron (MLP) and Support Vector Machine (SVM).

Table S5: Scikit–learn classifiers parameters for the highest values of F_1 score.

**Rotamer families**	**Reference**[a]	**Classifier**[b]	**Scikit-learn parameters**	**F1 score**
46	Sequence	NN	KNeighborsClassifier(2)	0.34
$\delta_{i-1}\delta_i\alpha\gamma$	Simple	NN	KNeighborsClassifier(1)	0.53
$\delta_{i-1}\delta_i\alpha$	Simple	NN	KNeighborsClassifier(1)	0.55
$\delta_{i-1}\delta_i\gamma$	Simple	NN	KNeighborsClassifier(1)	0.63
$\alpha\gamma$	Sequence	NN	KNeighborsClassifier(4)	0.59
$\delta_{i-1}\delta_i$	Simple	NN	KNeighborsClassifier(1)	0.77
A*_noA*	Sequence	NN	KNeighborsClassifier(1)	0.63
A _noA	Sequence	NN	KNeighborsClassifier(2)	0.61
46	Simple	DT	DecisionTreeClassifier(criterion='entropy', max_depth=None)	0.16
$\delta_{i-1}\delta_i\alpha\gamma$	Simple	DT	DecisionTreeClassifier(criterion='gini', max_depth=20)	0.43
$\delta_{i-1}\delta_i\alpha$	Simple	DT	DecisionTreeClassifier(criterion='gini', max_depth=None)	0.48
$\delta_{i-1}\delta_i\gamma$	Simple	DT	DecisionTreeClassifier(criterion='gini', max_depth=None)	0.61
$\alpha\gamma$	Sequence	DT	DecisionTreeClassifier(criterion='entropy', max_depth=20)	0.47
$\delta_{i-1}\delta_i$	Simple	DT	DecisionTreeClassifier(criterion='gini', max_depth=None)	0.73
A*_noA*	Sequence	DT	DecisionTreeClassifier(criterion='entropy', max_depth=20)	0.59
A _noA	Sequence	DT	DecisionTreeClassifier(criterion='entropy', max_depth=20)	0.52
46	Simple	RF	RandomForestClassifier(criterion='gini', max_depth=20, n_estimators=10, max_features=1)	0.24
$\delta_{i-1}\delta_i\alpha\gamma$	Simple	RF	RandomForestClassifier(criterion='entropy', max_depth=20, n_estimators=10, max_features=1)	0.49
$\delta_{i-1}\delta_i\alpha$	Puckering	RF	RandomForestClassifier(criterion='entropy', max_depth=20, n_estimators=10, max_features=1)	0.49
$\delta_{i-1}\delta_i\gamma$	Puckering	RF	RandomForestClassifier(criterion='entropy', max_depth=20, n_estimators=10, max_features='auto')	0.64
$\alpha\gamma$	C_mean	RF	RandomForestClassifier(criterion='entropy', max_depth=20, n_estimators=10, max_features=1)	0.53
$\delta_{i-1}\delta_i$	Puckering	RF	RandomForestClassifier(criterion='gini', max_depth=20, n_estimators=10, max_features=1)	0.74
A*_noA*	Sequence	RF	RandomForestClassifier(criterion='entropy', max_depth=20, n_estimators=10, max_features='auto')	0.61
A _noA	Sequence	RF	RandomForestClassifier(criterion='entropy', max_depth=20, n_estimators=10, max_features='auto')	0.49
46	Puckering	MLP	MLPClassifier(alpha=0.0001, max_iter=500, solver='lbfgs')	0.11
$\delta_{i-1}\delta_i\alpha\gamma$	Sequence	MLP	MLPClassifier(alpha=0.0001, max_iter=750, solver='sgd')	0.55
$\delta_{i-1}\delta_i\alpha$	Puckering	MLP	MLPClassifier(alpha=0.0001, max_iter=1000, solver='sgd')	0.52
$\delta_{i-1}\delta_i\gamma$	C_mean	MLP	MLPClassifier(alpha=0.0001, max_iter=500, solver='lbfgs')	0.62
$\alpha\gamma$	Sequence	MLP	MLPClassifier(alpha=0.0001, max_iter=1000, solver='sgd')	0.63
$\delta_{i-1}\delta_i$	Simple	MLP	MLPClassifier(alpha=0.0001, max_iter=500, solver='adam')	0.72
A*_noA*	C_opt	MLP	MLPClassifier(alpha=0.0001, max_iter=500, solver='lbfgs')	0.44
A _noA	C_opt	MLP	MLPClassifier(alpha=0.0001, max_iter=500, solver='lbfgs')	0.41
46	Sequence	SVM	SVC(kernel='linear', C=0.025)	0.31
$\delta_{i-1}\delta_i\alpha\gamma$	Simple	SVM	SVC(kernel='rbf', C=0.025)	0.55
$\delta_{i-1}\delta_i\alpha$	C_mean	SVM	SVC(kernel='rbf', C=0.025)	0.57
$\delta_{i-1}\delta_i\gamma$	C_mean	SVM	SVC(kernel='linear', C=0.025)	0.6
$\alpha\gamma$	Simple	SVM	SVC(kernel='rbf', C=0.025)	0.62
$\delta_{i-1}\delta_i$	Simple	SVM	SVC(kernel='linear', C=0.025)	0.75
A*_noA*	Simple	SVM	SVC(kernel='rbf', C=2.0)	0.36
A _noA	Simple	SVM	SVC(kernel='linear', C=0.5)	0.62

a **Reference** refers to the effective references used to obtain the theoretical $^{13}C'$ chemical shifts (see Section 4).
b **Classifier**: Nearest Neighbor (NN), Decision Tree (DT), Random Forest (RF), Multi-Layer Perceptron (MLP) and Support Vector Machine (SVM)

References

[1] Richardson,J.S., Schneider,B., Murray,L.W., Kapral,G.J., Immormino,R.M., Headd,J.J., Richardson,D.C., Ham,D., Hershkovits,E., Williams,L.D. et al. (2008) RNA backbone: Consensus all-angle conformers and modular string nomenclature (an RNA Ontology Consortium contribution). *RNA*, **14**, 465481.

[2] Salvatier,J., Wiecki,T.V. and Fonnesbeck,C. (2016) Probabilistic programming in Python using PyMC3. *PeerJ Computer Science* 2:e55.
